# A new integrated framework for the identification of potential virus–drug associations

**DOI:** 10.3389/fmicb.2023.1179414

**Published:** 2023-08-22

**Authors:** Jia Qu, Zihao Song, Xiaolong Cheng, Zhibin Jiang, Jie Zhou

**Affiliations:** ^1^School of Computer Science and Artificial Intelligence, Changzhou University, Changzhou, Jiangsu, China; ^2^School of Computer Science and Engineering, Shaoxing University, Shaoxing, Zhejiang, China

**Keywords:** drug, virus, association prediction, matrix decomposition, Bounded Nuclear Norm Regularization, ensemble learning

## Abstract

**Introduction:**

With the increasingly serious problem of antiviral drug resistance, drug repurposing offers a time-efficient and cost-effective way to find potential therapeutic agents for disease. Computational models have the ability to quickly predict potential reusable drug candidates to treat diseases.

**Methods:**

In this study, two matrix decomposition-based methods, i.e., Matrix Decomposition with Heterogeneous Graph Inference (MDHGI) and Bounded Nuclear Norm Regularization (BNNR), were integrated to predict anti-viral drugs. Moreover, global leave-one-out cross-validation (LOOCV), local LOOCV, and 5-fold cross-validation were implemented to evaluate the performance of the proposed model based on datasets of DrugVirus that consist of 933 known associations between 175 drugs and 95 viruses.

**Results:**

The results showed that the area under the receiver operating characteristics curve (AUC) of global LOOCV and local LOOCV are 0.9035 and 0.8786, respectively. The average AUC and the standard deviation of the 5-fold cross-validation for DrugVirus datasets are 0.8856 ± 0.0032. We further implemented cross-validation based on MDAD and aBiofilm, respectively, to evaluate the performance of the model. In particle, MDAD (aBiofilm) dataset contains 2,470 (2,884) known associations between 1,373 (1,470) drugs and 173 (140) microbes. In addition, two types of case studies were carried out further to verify the effectiveness of the model based on the DrugVirus and MDAD datasets. The results of the case studies supported the effectiveness of MHBVDA in identifying potential virus-drug associations as well as predicting potential drugs for new microbes.

## Introduction

The lives of humans and other higher animals are closely related to microbial communities that include bacteria, archaea, viruses, fungi, and protozoa (Sommer and Bäckhed, 2013). On the earth, the number of viruses is dozens of times higher than that of bacteria (Lawrence et al., 2009). No surprise, viruses are widely distributed in the environment and biological tissues, including water, soil, and human bodies (Wigington et al., 2016). By infecting host cells and proliferating in host cells, viruses can cause a variety of human diseases (Maarouf et al., 2018). For example, the spike protein of Severe Acute Respiratory Syndrome Coronavirus 2 (SARS-CoV-2) mediates SARS-CoV-2 entry into cells and can infect bronchial epithelial cells, pneumocytes, and upper respiratory tract cells in humans (Shang et al., 2020). Thus, SARS-CoV-2 can cause respiratory lesions and lung injuries (V'kovski et al., 2021). Besides, the Ebola virus (EBOV) can enter the body through broken skin or *via* mucosal surfaces, which further results in EBOV infections (Dowell et al., 1999). EBOV infections are able to cause fever, mucosal hemorrhages, and even death (Rivera and Messaoudi, 2016).

As we all know, the outbreak of SARS-CoV-2 in Wuhan, China, in December 2019 posed an enormous public health threat and a pandemic threat (Lu et al., 2020). Hoffmann et al. (2020) found camostat mesylate could prevent SARS-CoV-2 from entering the host cell by inhibiting the serine protease TMPRSS2. Moreover, ZIKV can cause serious neurological complications, such as Guillain-Barré syndrome and meningoencephalitis (Cao-Lormeau et al., 2016). The study by Zhou et al. (2017) showed that hippeastrine hydrobromide and amodiaquine dihydrochloride dihydrate could inhibit ZIKV infection in human cortical neural progenitor cells. Obviously, there is an urgent need to find effective antiviral drugs. Identifying virus-drug associations not only helps understand the mechanisms of interactions between viruses and drugs but also contributes to the discovery of potential antiviral drugs.

Drug discovery, one of the main goals of pharmaceutical sciences, is an interdisciplinary field that includes basic sciences such as biology, chemistry, physics, and statistics (Liu et al., 2016). There are currently two main challenges to drug development. On the one hand, the development of a drug usually takes a long time from the start of development to obtain marketing approval (Parvathaneni et al., 2019). On the other hand, more and more cases show that drug resistance has begun to appear, posing a serious threat to human health (Ramirez et al., 2016). For example, Acyclovir (ACV) is an effective drug for the treatment of herpes simplex virus (HSV) infection (Piret and Boivin, 2016). However, for serious infections in immunocompromised patients, long-term use of ACV can cause the development of drug resistance (Jiang et al., 2016). The emergence of ACV resistance made the treatment of HSV infection more difficult (Jiang et al., 2016). In order to solve these issues, drug combination therapies have been used to treat multiple complex diseases such as cancer and hypertension (Wang et al., 2017). In addition, drug repurposing, also called drug repositioning, is based on the idea of using existing drugs to treat emerging and challenging diseases (Pushpakom et al., 2019). For drug combination therapies and drug repositioning, it is crucial to identify virus–drug associations.

Identifying virus-related drugs can not only help understand the mechanisms of interactions between viruses and drugs but also contribute to the discovery of potential antiviral drugs. Since traditional laboratory methods are time-consuming and costly, numerous computational models have been proposed to predict potential associations between viruses and drugs (Xu et al., 2023). It is extremely urgent to develop efficient calculation algorithms to predict potential virus–drug associations. Recently, some computational models have been proposed to effectively identify the potential associations between drugs and viruses. For example, Peng et al. (2020) proposed a virus–drug association prediction model of VDA-RLSBN based on regularized least squared (RLS) classifier and bipartite local model. For a given virus, its related drugs can be predicted by RLS based on original association information and the kernel matrix that can be obtained from virus similarity. In the same way, based on drug similarity, drug-related viruses can be identified by RLS. At last, an integrated strategy was implemented to integrate the two predicted scores. Besides, Zhou et al. (2020) developed a computational model of virus–drug association prediction based on the KATZ method (VDA-KATZ) to identify potential antiviral drugs against SARS-CoV-2. KATZ is a network-based method that calculates the similarity of nodes by considering step size and the number of walks between nodes in heterogeneous networks (Katz, 1953). Moreover, Long et al. (2020) proposed a model of a graph convolutional network (GCN) for predicting human Microbe-Drug Associations (GCNMDA). In the model, based on drug and microbe similarity, random walk with restart was implemented to effectively capture valuable features for drugs and microbes, respectively. Then, GCN was used to learn representations for drugs and microbes. At last, an attention mechanism was designed in the conditional random field layer for aggregating representations of neighborhoods. In 2021, Long and Luo (2021) also presented a model of Heterogeneous Network Embedding Representation for Microbe-Drug Association prediction (HNERMDA). First, drug–drug interactions, microbe–microbe interactions, and known microbe–drug associations were integrated to build a heterogeneous network. Second, metapath2vec was adopted to study low-dimensional embedding representations for both drugs and microbes. Finally, a bipartite network recommendation algorithm was carried out to predict new microbe–drug associations. In addition, in 2022, Ma and Liu (2022) developed a Weighted Hypergraph Generalized Matrix Factorization model for Microbe-Drug Association prediction. In this model, microbe and drug hypergraph were constructed using K-nearest neighbors based on a variety of biological data. Then, microbe-weighted and drug-weighted hypergraphs were calculated by the method of simplicity volume based on microbe and drug hypergraphs. At last, potential microbe–drug associations can be inferred by the generalized matrix factorization based on the microbe-weighted and drug-weighted hypergraphs. In 2023, Huang et al. (2023) proposed a novel prediction framework based on the Graph Normalized Auto-Encoder to predict Microbe-Drug Associations (GNAEMDA). First, multi-modal attributes of microbes and drugs were constructed using multiple similarity data for microbes and drugs. Subsequently, the microbe–drug association network and multi-modal attributes of microbes and drugs were used as the input of the graph normalized convolutional network (GNCN). Second, the node embedding matrix of the microbe-drug association was calculated by GNCN. Finally, the potential microbe–drug associations were predicted based on the microbe–drug association graphs by using the inner product decoder. In the same year, Huang et al. (2023) proposed a variational GNAEMDA model (VGNAEMDA) for microbe–drug associations. Different from GNAEMDA, a residual module was added to the GNCN (RGNCN). The node embedding matrix of the graph for microbe–drug association was calculated by using GNCN and RGNCN. Subsequently, potential microbe–drug associations were identified by using inner product decoder. Moreover, Tian et al. (2023) proposed a novel method that employs Structure-enhanced Contrastive learning and Self-paced negative sampling strategy to identify potential Microbe-Drug Associations. In this model, based on the connection mode of different nodes in the MDA networks, two types of meta-path-inducted networks for microbes, and two types of meta-path-induced networks for drugs were constructed, respectively. Subsequently, the node embedding representations of integrated microbe similarity networks, integrated drug similarity networks, two types of meta-path-induced network for microbes, and two types of meta-path-induced networks for drugs were learned through GCNs, respectively. For microbes (drugs), based on different microbe (drug) meta-path-induced networks, the final embeddings of microbes (drugs) were calculated by semantic level attention. Moreover, the structure-enhanced contrastive strategy employed the final embedding of microbes (drugs) calculated from different microbe meta-path-induced networks to enhance the node embedding representations of microbes (drugs) learned from the integrated microbe (drug) similarity network as the final node representations of microbes (drugs). Furthermore, the values for all the candidate negative microbe–drug association pairs were calculated by the multilayer perceptron (MLP) classifier. At last, the final embedded representations of microbes and drugs were input to the MLP decoder, and then the microbe–drug association probabilities could be obtained.

In this study, we developed an integrated model, named MHBVDA, to identify potential virus–drug associations based on Matrix Decomposition with Heterogeneous Graph Inference (MDHGI) and Bounded Nuclear Norm Regularization (BNNR). In MDHGI, based on the new adjacency matrix of virus-drug associations acquired from matrix decomposition by using the sparse learning method, a two-layer heterogeneous graph inference was constructed to predict potential virus-drug associations. In BNNR, based on the matrix built by integrating multi-source data, a target equation that completed this matrix was constructed by minimizing its nuclear norm. Then, the alternating direction method of multipliers was carried out to minimize the nuclear norm and gain predicted scores. At last, an ensemble learning strategy was employed to integrate the two different prediction models. To evaluate the performance of MHBVDA, global leave-one-out cross-validation (LOOCV) and local LOOCV as well as 5-fold cross-validation were implemented based on the dataset of DrugVirus (Long et al., 2020). Experimental results showed that the area under the receiver operating characteristics curves (AUC) of global LOOCV and local LOOCV are 0.9035 and 0.8786, respectively. The average AUC and the standard deviation of the 5-fold cross-validation are 0.8856 ± 0.0032. In order to evaluate the applicability of the model in other datasets, we also implemented LOOCV and 5-fold cross-validation on the other two datasets of MDAD (Sun et al., 2018) and aBiofilm (Rajput et al., 2018). At last, compared with the recent six models, MHBVDA obtained better performance based on the datasets of MDAD and aBiofilm, respectively. Furthermore, two types of case studies were implemented based on DrugVirus and MDAD datasets to evaluate the performance of the MHBVDA. In the case studies, the results showed that 19, 25, 24, and 22 out of the top 50 predicted drugs for ZIKV, SARS-CoV-2, HIV-1, and *Pseudomonas aeruginosa* were confirmed, respectively. MHBVDA could be a promising tool for predicting potential virus-drug associations.

## Materials and methods

### Dataset

#### Virus–drug association

The dataset of known virus–drug association information used in this model was collected from the DrugVirus database (Long et al., 2020). The dataset includes 933 known virus–drug associations between 175 drugs and 95 viruses. The adjacency matrix *A*(*nd*×*nv*) was further constructed to store virus–drug association information. In the matrix of *A*, *nd* represents the number of drugs and *nv* denotes the number of viruses. If the drug is *d*_*i*_ related to virus *v*_*j*_, the entity *A*(*i, j*) is 1, otherwise 0.


(1) $$A(i,j)=\{\begin{array}{ll} 1, & \text{ if drug }d_{i}\text{ associated with virus }v_{j}\\ 0, & \text{ otherwise} \end{array}$$

#### Drug chemical structure similarity

A chemical structure search server of SIMCOMP (http://www.genome.jp/tools/simcomp/) was used to calculate the drug chemical structure similarity (Hattori et al., 2003; Kanehisa et al., 2008, 2019). SIMCOMP treats drugs as graphs and computes a similarity score between the two drugs based on their graphs. First, we downloaded MOL files of drugs (compounds) from the KEGG DRUG Database (https://www.genome.jp/kegg/drug/). Then, we imported MOL files of drugs into SIMCOMP that can compute a global similarity based on the common substructures of two drugs (Hattori et al., 2003). The matrix *SS*1 was built to save chemical structure similarity and entity *SS*1(*i, j*) represented the chemical structure similarity between drug *d*_*i*_ and drug *d*_j_.

#### Drug side effect similarity

Data of drug side effects used in this study were obtained from SIDER that is a drug side effect database (http://sideeffects.embl.de/) (Kuhn et al., 2016). We used *M*(*i*) to represent the set of side effects related to drug *d*_j_ and *M*(*j*) to denote the set of side effects related to drug *d*_j_. The entity *SS*2(*i, j*) was used to represent the side effect similarity between drug *d*_*i*_ and drug *d*_j_. If two drugs share more side effects, their side effects similarity is more similar. If they have no common side effects, the value of side effects similarity is 0. Finally, Jaccard score was employed to calculate the similarity of the drugs side effects (Gottlieb et al., 2011). The calculation formula is as follows.


(2) $$\begin{array}{l} SS2\left(i,j\right)=\text{Jaccard score}=\frac{\left|M\left(i\right)\cap M\left(j\right)\right|}{\left|M\left(i\right)\cup M\left(j\right)\right|} \end{array}$$

#### Virus sequence similarity

In this study, we downloaded the complete genome sequences of 95 viruses in FASTA format from the National Center for Biotechnology Information (NCBI) (https://www.ncbi.nlm.nih.gov/). Subsequently, we used the multiple sequence alignment software MAFFT to align the complete genome sequence of the viruses (Katoh et al., 2002). After aligning the viral complete genome sequence using MAFFT, we employed BioEdit, a gratis sequence analysis tool, to obtain the virus sequence similarity matrix (Tippmann, 2004). Based on the concept that the more sequences two viruses share, the more similar they are. If two viruses have no common sequences, their sequence similarity value is 0. Here, the matrix *MV* was defined to store virus sequence similarity and *MV*(*v*_*i*_, *v*_*j*_) represented the sequence similarity between virus *v*_*i*_ and virus *v*_*j*_. If a virus has no complete genome sequence in NCBI, the sequence similarity value between the virus and other viruses is set to 0.

#### Gaussian interaction profile kernel similarity for drugs and viruses

Based on the idea that similar viruses (drugs) are associated with similar drugs (viruses), a Gaussian interaction profile kernel similarity for drugs and viruses was constructed in the model (Van Laarhoven et al., 2011). For the virus–drug association matrix *A*, we used *IV*(*d*_*i*_) to represent the *i-th* row vector and *IV*(*v*_*j*_) to indicate the *j-th* column vector. The Gaussian interaction profile kernel similarity for viruses and drugs can be calculated as equations (3) and (4), respectively.


(3) $$\begin{array}{l} GV\left(v_{i},v_{j}\right)=\exp\left(-\beta_{v}{||IV\left(v_{i}\right)-IV\left(v_{j}\right)||}^{2}\right) \end{array}$$

(4) $$\begin{array}{l} GD\left(d_{i},d_{j}\right)=\exp\left(-\beta_{d}{||IV\left(d_{i}\right)-IV\left(d_{j}\right)||}^{2}\right) \end{array}$$

where ${\left||IV\left(v_{i}\right)-IV\left(v_{j}\right)|\right|}^{2}$ can be regarded as the square of the Euclidean distance between feature vector and feature vector *IV*(*v*_*j*_), and ${\left||IV\left(d_{i}\right)-IV\left(d_{j}\right)|\right|}^{2}$ can be regarded as the square of the Euclidean distance between feature vector and feature vector *IV*(*d*_*j*_); the parameters β_*v*_ and β_*d*_ were defined as follows:


(5) $$\beta_{v}\text{=}\beta_{v^{\prime }}/\left(\frac{1}{nv}{\sum_{i=1}^{nv}\left\|IV\left(v_{i}\right)\right\|}^{2}\right)$$

(6) $$\beta_{d}\text{=}\beta_{{\text{d}}^{\prime }}/\left(\frac{1}{nd}{\sum_{i=1}^{nd}\left\|IV\left(d_{i}\right)\right\|}^{2}\right)$$

where ||•||^2^ is L2-norm and $\beta_{d}^{\prime }$ and $\beta_{m}^{\prime }$ are set as 1.

#### Integrated similarity for viruses and drugs

In order to obtain the integrated drug similarity, we integrated drug chemical structure similarity, drug side effect similarity, and the Gaussian interaction profile kernel similarity of the drug. If drugs *d*_*i*_ and *d*_*j*_ have chemical structural similarity or side effect similarity, the integrated drug similarity is the average of drug chemical structural similarity and drug side effect similarity. Otherwise, integrated drug similarity is equal to the value of the Gaussian interaction profile kernel similarity of the drug. The formula is as follows:


(7) $$SD(d_{i},d_{j})=\{\begin{array}{ll} \frac{SS1(d_{i},d_{j})+SS2(d_{i},d_{j})}{2} & d_{i}\text{ and }d_{j}\text{ have chemical structure or side effect similarity}\\ GD(d_{i},d_{j}) & \text{ otherwise} \end{array}$$

For virus similarity, we integrated the virus sequence similarity and the Gaussian interaction profile kernel similarity of virus for obtaining the integrated virus similarity. The formula is as follows:


(8) $$SV(v_{i},v_{j})=\{\begin{array}{ll} MV(v_{i},v_{j}) & v_{i}\text{ and }v_{j}\text{ have sequence similarity}\\ GV(v_{i},v_{j}) & \text{otherwise} \end{array}$$

### MHBVDA

**Figure 1:**
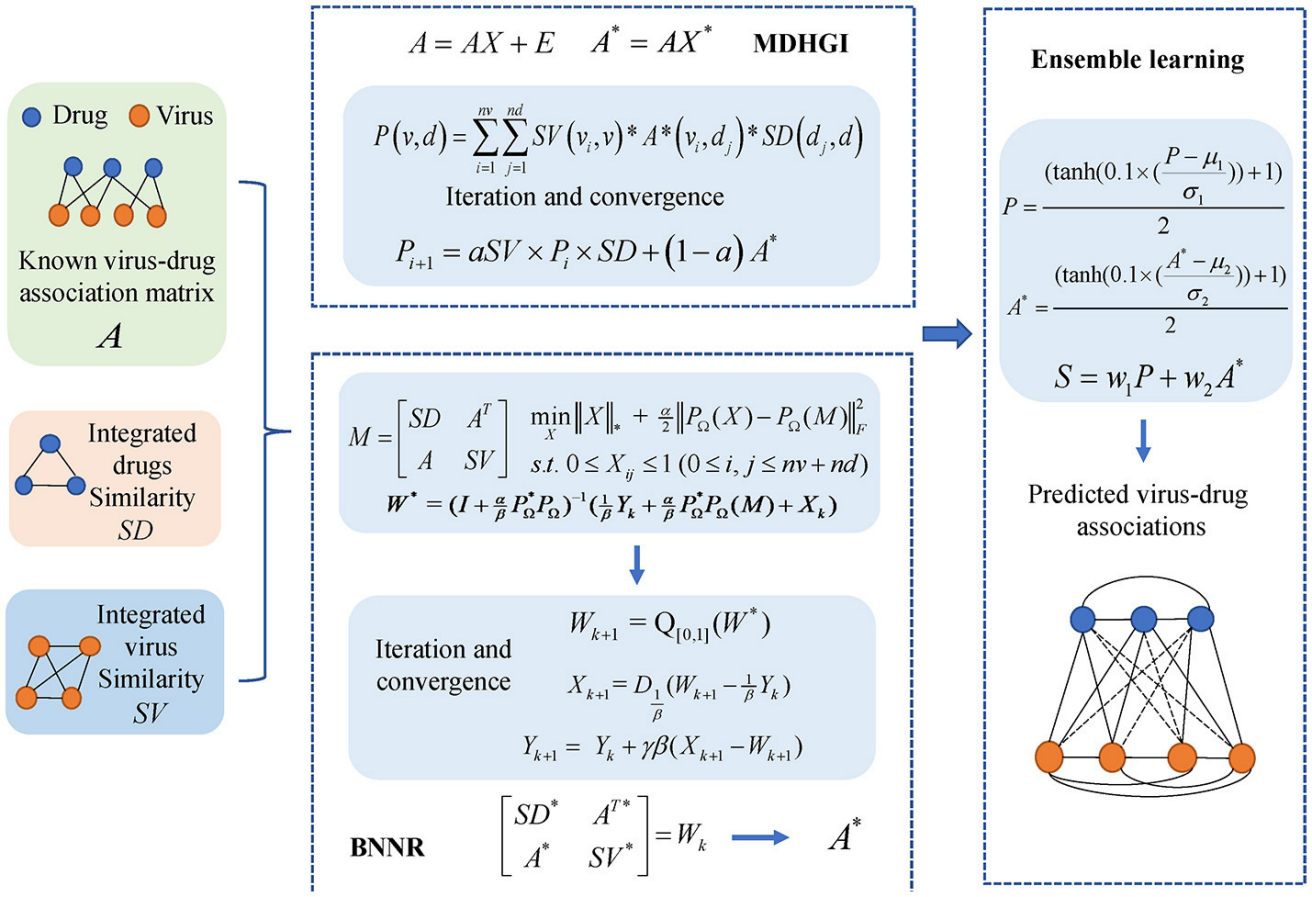
We constructed an integrated model, named MHBVDA, for predicting potential virus–drug association based on MDHGI (Chen et al., 2018b) and BNNR (Chen et al., 2021).

In this study, we constructed an integrated model, named MHBVD, for predicting potential virus–drug association based on MDHGI (Chen et al., 2018b) and BNNR (Chen et al., 2021). The flowchart of the MHBVDA is shown in Figure 1.

#### Matrix decomposition with heterogeneous graph inference

Some virus–drug associations used in the model may be redundant or missing. Therefore, we decomposed the adjacency matrix A of virus–drug associations into two portions. The first portion is a product of the original matrix and a low-rank matrix that includes non-redundant data. The second portion is a sparse matrix in which elements are mostly zero. Here, we used the nuclear norm for *X* to obtain a low-rank matrix and used sparse norm for *E* to gain a sparse matrix. The decomposition equation is as follows:


(9) $$\begin{array}{ll} \underset{X,E}{min}{\left\|X\right\|}_{*}+\alpha {\left\|E\right\|}_{2,1} & \;s.t.\;A=AX+E\\ {\left\|X\right\|}_{*}\text{=}\sum_{i}\sigma_{i}\left(i.e.,\sigma_{i}\text{ is the singular value of }X\right) & \\ {\left\|E\right\|}_{2,1}\text{=}\sum_{j=1}^{n}\sqrt{\sum_{i=1}^{n}{\left(E_{ij}\right)}^{2}} & \end{array}$$

In Equation (9), α is used to control the weights of *X* and *E*. Equation (9) can be rewritten as shown below:


(10) $$\underset{X,E,J}{min}{\left\|J\right\|}_{*}+\alpha {\left\|E\right\|}_{2,1}\;s.t.\;A=AX+E,X=J$$

In simple terms, Equation (10) could be regarded as a constraint and convex optimization problem. We employed inexact augmented Lagrange multipliers (IALM) (Meng et al., 2014) to solve the problem as follows:


(11) $$\begin{array}{l} L=\;{\left\|J\right\|}_{*}+\alpha {\left\|E\right\|}_{2,1}+tr\left(Y_{1}^{T}\left(A-AX-E\right)\right)+tr\left(Y_{2}^{T}\left(X-J\right)\right)\\ \;+\frac{\mu }{2}\left({\left\|A-AX-E\right\|}_{F}^{2}+{\left\|X-J\right\|}_{F}^{2}\right) \end{array}$$

In Equation (11), μ is the penalty parameter and μ≥0. We could obtain two solutions defined as X^*^ and E^*^ from Equation (11), and detailed solution steps are shown in Algorithm: IALM (see Table 1). Then, we built a new virus–drug association matrix A^*^ by using AX^*^. Subsequently, the potential probability of drugs associated with viruses could be predicted by incorporating the new virus–drug association matrix A^*^, integrated drug similarity *SD*, and integrated virus similarity *SV* into a heterogeneous graph and further using heterogeneous graph inference. We defined the potential association probability between virus *v* and drug as follows:


(12) $$\begin{array}{l} P\left(v,d\right)=\overset{nv}{\underset{i=1}{\sum }}\overset{nd}{\underset{j=1}{\sum }}SV\left(v_{i},v\right)*A^{*}\left(v_{i},d_{j}\right)*SD\left(d_{j},d\right) \end{array}$$

where *v*_*i*_ denotes *i*-*th* virus in DrugVirus dataset and *d*_*j*_ represents *j*-*th* drug in DrugVirus dataset.

**Table 1:** Computational procedures of the inexact augmented Lagrange multipliers (IALM) algorithm.

**Algorithm: IALM**
**Input:** known virus–drug adjacency matrix *A*, parameters α = 0.1
**Output:** X^*^ and E^*^
**Initialize:** $X=0,E=0,Y_{1}=0,Y_{2}=0,\mu =10^{-4},\underset{\mu }{\max}=10^{10},\;\rho =1.1$ **while** ${\left||A-AX-E|\right|}_{\infty }\geq \text{1}{\text{0}}^{-6}$ and ${\left||X-J|\right|}_{\infty }\geq \text{1}{\text{0}}^{-6}$ **do**
$(U,S,V)=\text{svd}((X+Y_{2}/\mu )\;\text{and}\;J=US_{\mu^{-1}}[S]V^{T}$
$X={\left(I+A^{T}A\right)}^{-1}\left(A^{T}A-A^{T}E+J+\left(A^{T}Y_{1}-Y_{2}\right)/\mu \right)$
$E=\text{argmin}\frac{\alpha }{\mu }||E|{|}_{2,1}+\frac{1}{2}||E-\left(A-AX+Y_{1}/\mu \right)|{|}_{F}^{2}$
*Y*_1_ = *Y*_1_+μ(*A*−*AX*−*E*), *Y*_2_ = *Y*_2_+μ(*X*−*J*)
$\mu =\min\left(\underset{\mu }{\max},\rho \mu \right)$
**end while**

Moreover, integrated drug similarity (*SD*) and integrated virus similarity (*SV*) are normalized to accelerate convergence of *p* as follows (Wang et al., 2013):


(13) $$\begin{array}{l} SV\left(v_{i},v_{j}\right)=\frac{SV\left(v_{i},v_{j}\right)}{\sqrt{\sum_{l=1}^{nv}SV\left(v_{i},v_{l}\right)}\sqrt{\sum_{l=1}^{nv}SV\left(v_{j},v_{l}\right)}} \end{array}$$

(14) $$\begin{array}{l} SD\left(d_{i},d_{j}\right)=\frac{SD\left(d_{i},d_{j}\right)}{\sqrt{\sum_{l=1}^{nd}SD\left(d_{i},d_{l}\right)}\sqrt{\sum_{l=1}^{nd}SD\left(d_{j},d_{l}\right)}} \end{array}$$

Furthermore, we used an iterative method to calculate potential association probability between drugs and viruses as Equation (15).


(15) $$\begin{array}{l} P_{i+1}=aSV\times P_{i}\times SD+\left(1-a\right)A^{*} \end{array}$$

where *P*_*i*_ is equal to A^*^ when *i* is equal to 0. The value of the decay factor α was set to 0.4 (Wang et al., 2013). When the difference between *P*_*i*_ and *P*_*i*+1_ is < 10^−6^ calculated by L1 norm, the iteration was terminated.

#### Bounded Nuclear Norm Regularization

Moreover, we also used the matrix completion method of BNNR to predict potential virus–drug associations. We first constructed a heterogeneous graph of virus–drug similarity by integrating virus similarity, drug similarity, and known virus–drug associations. Subsequently, a target matrix is defined to denote a heterogeneous graph of virus–drug associations as follows:


(16) $$\begin{array}{l} M=\left[\begin{array}{cc} SD & A^{T}\\ A & SV \end{array}\right] \end{array}$$

The goal of defining *M* is to complete the unknown values in *A*. Assuming target matrix is low rank, matrix completion problem can be formulated as follows (Ramlatchan et al., 2018):


(17) $$\begin{array}{l} \begin{array}{c} \;\min\;rank\left(X\right)\\ s.t.\;P_{\Omega }\left(X\right)=P_{\Omega }\left(M\right) \end{array} \end{array}$$

where *M*∈*R*^(*nd*+*nv*) × (*nd*+*nv*)^ is the matrix to be completed, represents the number of virus, *nd* denotes the number of drug, *rank*(·) represents the rank function, Ω is a set of index pairs corresponding all known virus–drug associations in *M*, and *P*_Ω_ is a projection operator onto Ω.


(18) $${\left(P_{\Omega }(X)\right)}_{ij}=\{\begin{array}{l} X_{ij},\;(i,j)\in \Omega \\ 0,\;(i,j)\notin \Omega \end{array}$$

However, rank minimization problem is NP-hard and rank function in Equation (21) is non-convex (Sun and Dai, 2015). Based on pervious study (Candes and Recht, 2013), Equation (21) can be relaxed as shown below:


(19) $$\begin{array}{c} \;\underset{X}{\min}{\left\|X\right\|}_{*}\\ s.t.\;P_{\Omega }(X)=P_{\Omega }(M) \end{array}$$

where ||*X*||_*_ is nuclear norm of *X*.

Since data of virus and drug may exist noise, we reconstructed the matrix completion model to tolerate noise as Equation (20) (Candes and Plan, 2010).


(20) $$\begin{array}{c} \underset{X}{\min}{\left\|X\right\|}_{*}\\ s.t.\;{\left\|P_{\Omega }(X)-P_{\Omega }(M)\right\|}_{F}\leq \in \end{array}$$

where ∈ denotes the noise level and || · ||_*F*_ indicates Frobenius norm.

As the noise level is unknown, selecting an appropriate parameter is difficult (Chen et al., 2012). Here, we used soft regularization term to solve the problem (Hu et al., 2012). Moreover, a bounded constraint is added to Equation (19) for predicted virus-drug associations with scores between 0 and 1, with practical meaning. Thus, a bound nuclear norm regularization method is presented to identify potential virus–drug associations in Equation (21).


(21) $$\begin{array}{l} \underset{X}{\min}{\left\|X\right\|}_{*}\text{+}\frac{\alpha }{2}{\left\|P_{\Omega }(X)-P_{\Omega }(M)\right\|}_{F}^{2}\\ s.t.\text{ 0}\leq X_{ij}\leq 1(\text{0}\leq i,j\leq nv+nd) \end{array}$$

where α was used to balance nuclear norm and error term, 0 ≤ *X*_*ij*_ ≤ 1(0 ≤ *i, j* ≤ *nv*+*nd*) represents all elements in *x*.

The alternating direction method of multipliers (ADMM) was used to solve Equation (21). Then, we introduced an auxiliary matrix *W* to optimize Equation (21) based on ADMM as follows:


(22) $$\begin{array}{c} \underset{X}{\min}{\left\|X\right\|}_{*}\text{ + }\frac{\alpha }{2}{\left\|P_{\Omega }(W)-P_{\Omega }(M)\right\|}_{F}^{2}\\ \;s.t.\;X=W\\ \text{0}\leq W_{ij}\leq 1\text{ (0}\leq i,j\leq nv+nd\text{)} \end{array}$$

Based on Equation (22), we can obtain the augmented Lagrangian function as follows:


(23) $$\begin{array}{l} L(W,X,Y,\alpha ,\beta )={\left\|X\right\|}_{*}\text{ + }\frac{\alpha }{2}{\left\|P_{\Omega }(W)-P_{\Omega }(M)\right\|}_{F}^{2}\\ \;+Tr(Y^{T}(X-W))+\frac{\beta }{2}{\left\|X-W\right\|}_{F}^{2} \end{array}$$

where *Y* is the Lagrange multiplier and β>0 represents penalty parameter. Then, we employed an iterative method to minimize function (23). At the *k*-*th* iteration, BNNR was used to compute *W*_*k*+1_, *X*_*k*+1_, and *Y*_*k*+1_ in turn. The specific process of computation can be found in the study written by Yang et al. (2019).

When the iteration is terminated, we can obtain matrix *W*_*k*_ as follows.


(24) $$\begin{array}{l} W_{k}=\left[\begin{array}{cc} SD^{*} & {A^{*}}^{T}\\ A^{*} & SV^{*} \end{array}\right] \end{array}$$

where A^*^ denotes predicted virus–drug association matrix.

In BNNR, the parameters of α, β, γ, *tol*_1_, *tol*_2_ are set as 1, 10, 1, 2 × 10^−3^, 10^−5^, respectively, according to the published literature (Yang et al., 2019). Computational procedure of the BNNR algorithm is shown in Table 2.

**Table 2:** Computational procedures of the Bounded Nuclear Norm Regularization algorithm.

**Algorithm: BNNR**
**Input:** The virus similarity matrix *SV*∈ℝ^*nv*×*nv*^, the drug similarity matrix *SD*∈ℝ^*nd*×*nd*^, the virus–drug association matrix *A*∈ℝ^*nd*×*nv*^, parameters α, β, *tol*_1_, *tol*_2_.
**Output:** The predicted association matrix of virus–drug A^*^.
**Initialize:** $X_{1}=P_{\Omega }\left(M\right),\;W_{1}=X_{1},\;Y_{1}=X_{1},\;\gamma \text{=1,}k=1,\;M=\left[\begin{array}{cc} SD & A^{T}\\ A & SV \end{array}\right];$
**while** $d1_{k+1}=\frac{{\left||X_{k+1}-X_{k}|\right|}_{F}}{{\left||X_{k}|\right|}_{F}}>tol_{1},\;d2_{k+1}=\frac{\left|d1_{k+1}-d1_{k}\right|}{\max\left\{|d1_{k}|,1\right\}}>tol_{2}$ **do**
$W^{*}\text{=}{\left(I+\frac{\alpha }{\beta }P_{\Omega }^{*}P_{\Omega }\right)}^{-1}\left(\frac{1}{\beta }Y_{k}+\frac{\alpha }{\beta }P_{\Omega }^{*}P_{\Omega }\left(M\right)+X_{k}\right)$
$W_{k+1}\text{=}{\text{Q}}_{\left[0,1\right]}\left(W^{*}\right)$;
$X_{k+1}=D_{\frac{1}{\beta }}\left(W_{k+1}-\frac{1}{\beta }Y_{k}\right)$;
*Y*_*k*+1_ = *Y*_*k*_+γβ(*X*_*k*+1_−*W*_*k*+1_);
*k* = *k*+1;
**end while**
$\left[\begin{array}{cc} SD^{*} & {A^{T}}^{*}\\ A^{*} & SV^{*} \end{array}\right]=W_{k}$;
**return** A^*^.

#### Ensemble learning

Because the generalization ability of a single predictor may be weak, ensemble learning is usually employed to integrate weak predictors to achieve stronger predictors (Polikar, 2006). Over the last couple of decades, Ensemble learning has been successfully applied in many fields including data stream classification, feature selection, and association prediction in bioinformatics (Gomes et al., 2017; Chen et al., 2018c; Lin et al., 2019). In this study, we employed the ensemble learning method to integrate MDHGI and BNNR for predicting potential virus–drug associations. To keep the predicted scores within 0 to 1, we normalized the scores obtained by MDHGI and BNNR as follows:


(25) $$\begin{array}{l} P=\frac{\left(\tanh\left(0.1\times \left(\frac{P-\mu_{1}}{\sigma_{1}}\right)\right)+1\right)}{2} \end{array}$$

(26) $$\begin{array}{l} A^{\text{*}}=\frac{\left(\tanh\left(0.1\times \left(\frac{A^{*}-\mu_{2}}{\sigma_{2}}\right)\right)+1\right)}{2} \end{array}$$

where μ_1_ and σ_1_ are mean and standard deviation obtained by the MDHGI, μ_2_ and σ_2_ are mean and standard deviation obtained by the BNNR. Then, we allocated different weights for MDHGI and BNNR to obtain better prediction performance. Finally, *S* was created to save the final score matrix of potential virus–drug associations, which can be described as follows:


(27) $$\begin{array}{l} S=w_{1}P+w_{2}A^{*} \end{array}$$

where *w*_1_ represents weight for MDHGI and *w*_2_ denotes weight for BNNR. The sum of *w*_1_ and *w*_2_ is equal to 1.

## Results

### Performance evaluation

#### Comparison with other baseline methods under DrugVirus dataset

**Figure 2:**
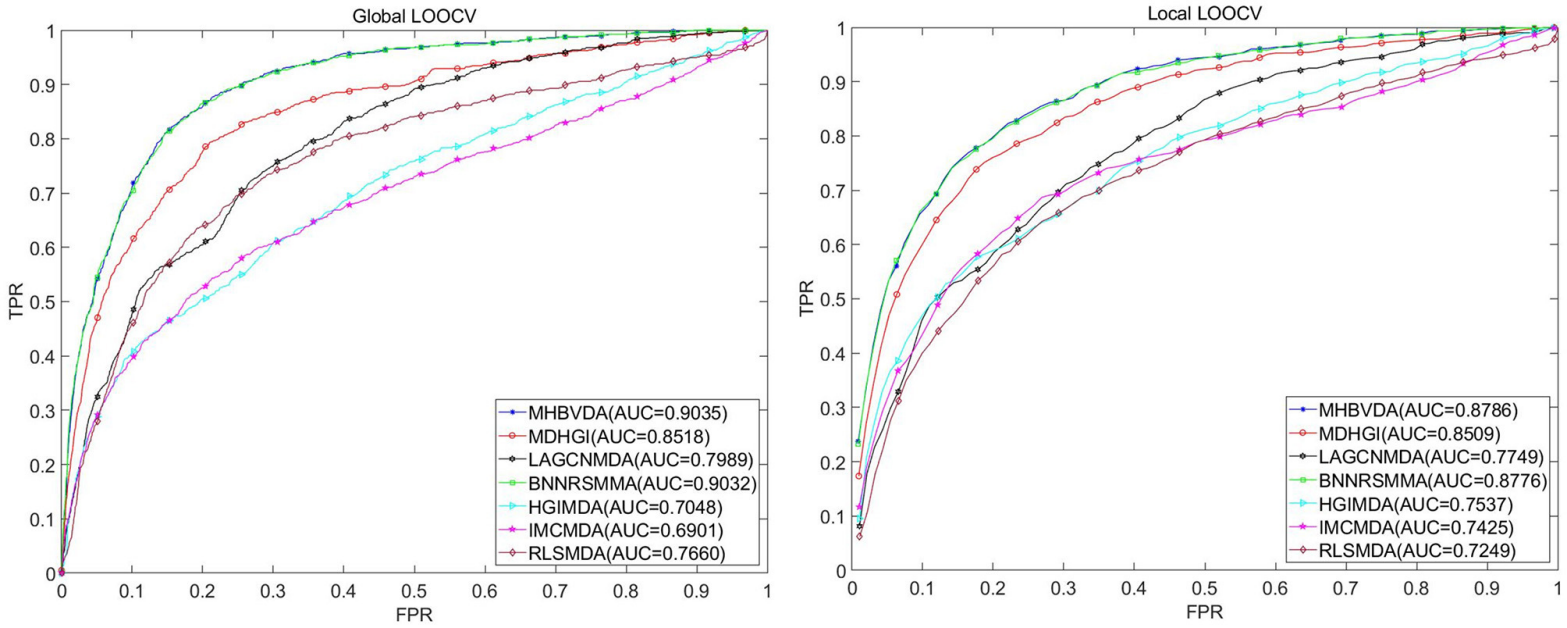
Performance comparison between MHBVDA and previous five association prediction models (MDHGIMDA, LAGCNMDA, BNNRSMMA, HGIMDA, IMCMDA, and RLSMDA) in AUC values of global LOOCV **(left)** and local LOOCV **(right)** based on the DrugVirus dataset.

In this study, we used global LOOCV, local LOOCV, and 5-fold cross-validation to evaluate the performance of MHBVDA based on the DrugVirus dataset that contains 933 known virus–drug associations between 175 drugs and 95 viruses. In the global LOOCV, each known virus–drug association was regarded as a test sample in turn; the remaining known virus–drug associations were used as training samples, and all unknown virus–drug pairs were regarded as candidate samples. However, in the local LOOCV, the candidate samples only included these virus–drug pairs where the virus had no known association with the investigated drug in the test samples. Then, we would obtain the ranking of test samples by comparing the score of each test sample with the scores of all candidate samples. We considered the MHBVDA successful in predicting test samples once the ranking of the test sample surpassed the pre-determined threshold. Furthermore, we drew the receiver operating characteristics (ROC) curve by plotting the true positive rate (TPR, sensitivity) against the false positive rate (FPR, 1-specificity) at different thresholds. Sensitivity indicates the percentage of test samples ranked over the given threshold, while specificity denotes the percentage of negative virus–drug associations whose ranking was lower than the given threshold. AUC equal to 1 indicates that the model has perfect prediction performance, while AUC equal to 0.5 means that the model's prediction is random. For DrugVirus, the result showed that MHBVDA obtained an AUC of 0.9035 in global LOOCV. Then, we compared the performance of MHBVDA with the other six classical models: RLSMDA (Chen and Yan, 2014), HGIMDA (Chen et al., 2016), IMCMDA (Chen et al., 2018a), MDHGI (Chen et al., 2018b), BNNRSMMA (Chen et al., 2021), and LAGCNMDA (Yu et al., 2021). The evaluation result showed that the AUCs of HGIMDA (0.7048), IMCMDA (0.6901), RLSMDA (0.7660), BNNRSMMA (0.9032), MDHGI (0.8518), and LAGCN (0.7989) are less than MHBVDA (see Figure 2). In the local LOOCV, MHBVDA derived better performance with an AUC of 0.8786 than HGIMDA (0.7537), IMCMDA (0.7425), RLSMDA (0.7249), BNNRSMMA (0.8776), MDHGI (0.8509), and LAGCNMDA (0.7749) (see Figure 2).

For 5-fold cross-validation, we randomly divided all known virus–drug association pairs into five subsets, of which four subsets contained 187 known virus–drug associations, respectively, whereas one contained 185 known virus–drug associations. Then, each subset was used as a test sample in turn, and the other four subsets were used as training samples. Similarly, all unknown virus–drug pairs were considered candidate samples. Subsequently, we obtained all test samples scores and the score ranking of each test sample by comparing scores between each test sample and all candidate samples. The prediction process of 5-fold cross-validation was repeated 100 times to avoid bias caused by random sample divisions. The results showed that the AUCs and standard deviations of HGIMDA (0.6995 ± 0.0024), IMCMDA (0.6776 ± 0.0034), RLSMDA (0.7238 ± 0.0246), BNNRSMMA (0.8830 ± 0.0034), MDHGI (0.8293 ± 0.0033), and LAGCNMDA (0.7999 ± 0.0016) are less than MHBVDA (0.8856 ± 0.0032) (see Table 3).

**Table 3:** Performance comparison between MHBVDA and previous five association prediction models (MDHGIMDA, LAGCNMDA, BNNRSMMA, HGIMDA, IMCMDA, and RLSMDA) in AUC values and standard deviations of 5-fold cross-validation based on datasets of DrugVirus, MDAD, and aBiofilm.

**Datasets**	**Performance**	**Methods**						
		**MHBVDA**	**HGIMDA**	**IMCMDA**	**RLSMDA**	**BNNRSMMA**	**MDHGI**	**LAGCNMDA**
DrugVirus	AUC	**0.8856**	0.6995	0.6776	0.7238	0.8830	0.8293	0.7999
	SD	**0.0032**	0.0024	0.0034	0.0246	0.0034	0.0033	0.0016
MDAD	AUC	**0.8857**	0.8152	0.7849	0.6571	0.7766	0.8153	0.8982
	SD	**0.0018**	0.0012	0.0025	0.0158	0.0018	0.0019	7.5868e-04
aBiofilm	AUC	**0.8871**	0.8412	0.7509	0.6338	0.7340	0.8176	0.9141
	SD	**0.0021**	0.0014	0.0073	0.0140	0.0073	0.0026	6.8556e-04

The bold values represent the performance of the proposed method MHBVDA for the identification of potential virus–drug associations on three different datasets.

The results showed that the AUCs of cross-validation for MHBVDA are higher than other compared algorithms based on the DrugVirus dataset. The outcome occurs because MHBVDA is an ensemble learning model based on BNNR and MDHGI. Therefore, the AUCs of cross-validation for MHBVDA are higher than those of HGIMDA, BNNRSMMA, and MDHGI. In addition, because the generalization ability of individual predictors is poor, ensemble learning is usually used to integrate several predictors to obtain a stronger predictor. Not surprisingly, the AUCs of cross-validation for MHBVDA are higher than those of IMCMDA, RLSMDA, and LAGCNMDA. In particle, though deep neural networks are powerful, it is well known that a huge amount of training data is usually required for training. Therefore, limited amounts of samples in the DrugVirus dataset may lead to inferior performance of deep learning-based models of LAGCNMDA.

#### Comparison with other baseline methods under different datasets

**Figure 3:**
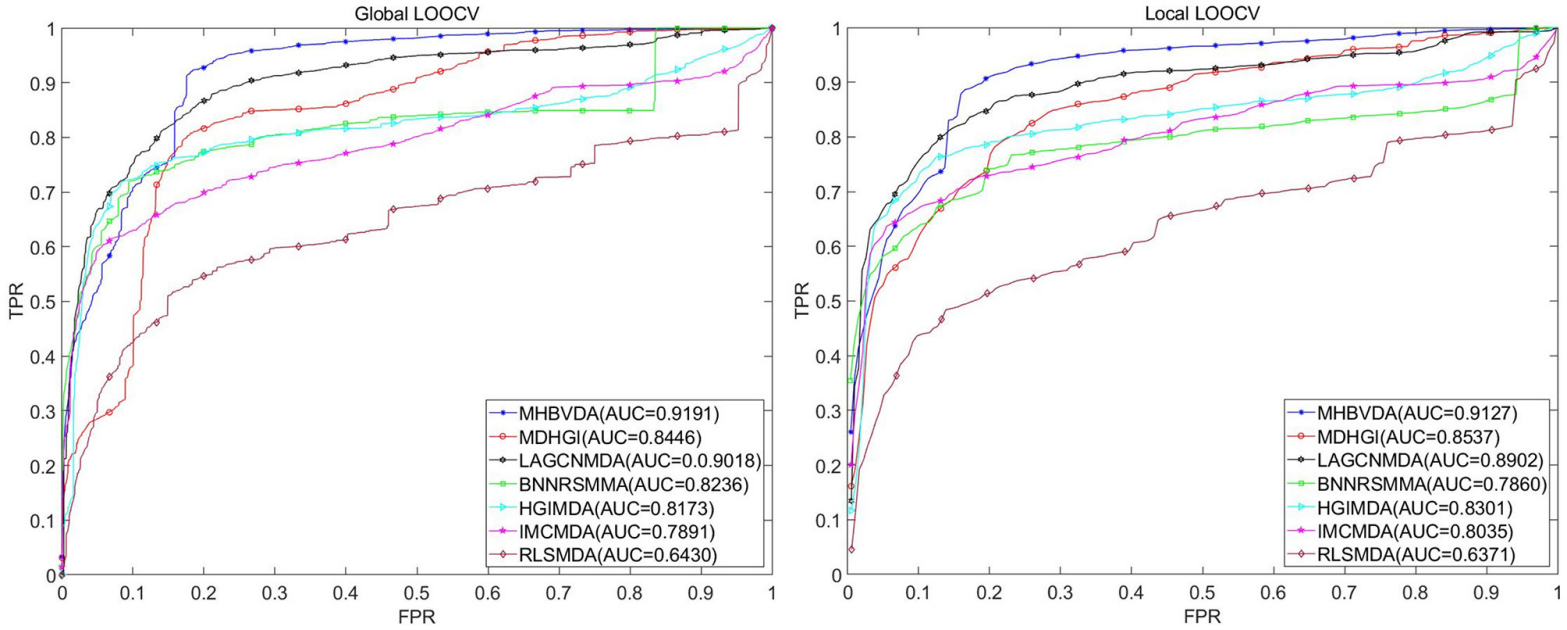
Performance comparison between MHBVDA and previous five association prediction models (MDHGIMDA, LAGCNMDA, BNNRSMMA, HGIMDA, IMCMDA, and RLSMDA) in AUC values of global LOOCV **(left)** and local LOOCV **(right)** based on the MDAD dataset.

As we all know, microbe communities, including viruses, bacteria, fungi, archaea, and protozoa, have a close relationship to human health (Sommer and Bäckhed, 2013). To further evaluate the predictive performance of MHBVDA, we carried out LOOCV and 5-fold cross-validation based on two other datasets of MDAD and aBiofilm, respectively. The MDAD dataset was constructed by collecting 2,470 microbe–drug associations between 173 microbes and 1,373 drugs from the MDAD database (https://github.com/Sun-Yazhou/MDAD) (Sun et al., 2018). For MDAD, in the global LOOCV, MHBVDA obtained an AUC of 0.9191, which is better than the AUCs of HGIMDA (0.8173), IMCMDA (0.7891), RLSMDA (0.6430), BNNRSMMA (0.8236), MDHGI (0.8446), and LAGCNMDA (0.9018) (see Figure 3). In the local LOOCV, MHBVDA derived better performance with AUC of 0.9127 than HGIMDA (0.8301), IMCMDA (0.8035), RLSMDA (0.6371), BNNRSMMA (0.7860), MDHGI (0.8537), and LAGCNMDA (0.8902) (see Figure 3). In the 5-fold cross-validation, AUCs and standard deviations of HGIMDA (0.8152 ± 0.0012), IMCMDA (0.7849 ± 0.0025), RLSMDA (0.6571 ± 0.0158), BNNRSMMA (0.7766 ± 0.0018), and MDHGI (0.8153 ± 0.0019) are less than MHBVDA (0.8857 ± 0.0018) (see Table 3). Although the AUCs and standard deviations of LAGCNMDA (0.8982 ± 7.5868e-04) are higher than those of MHBVDA, the global LOOCV and local LOOCV results of MHBVDA are higher than those of LAGCNMDA.

**Figure 4:**
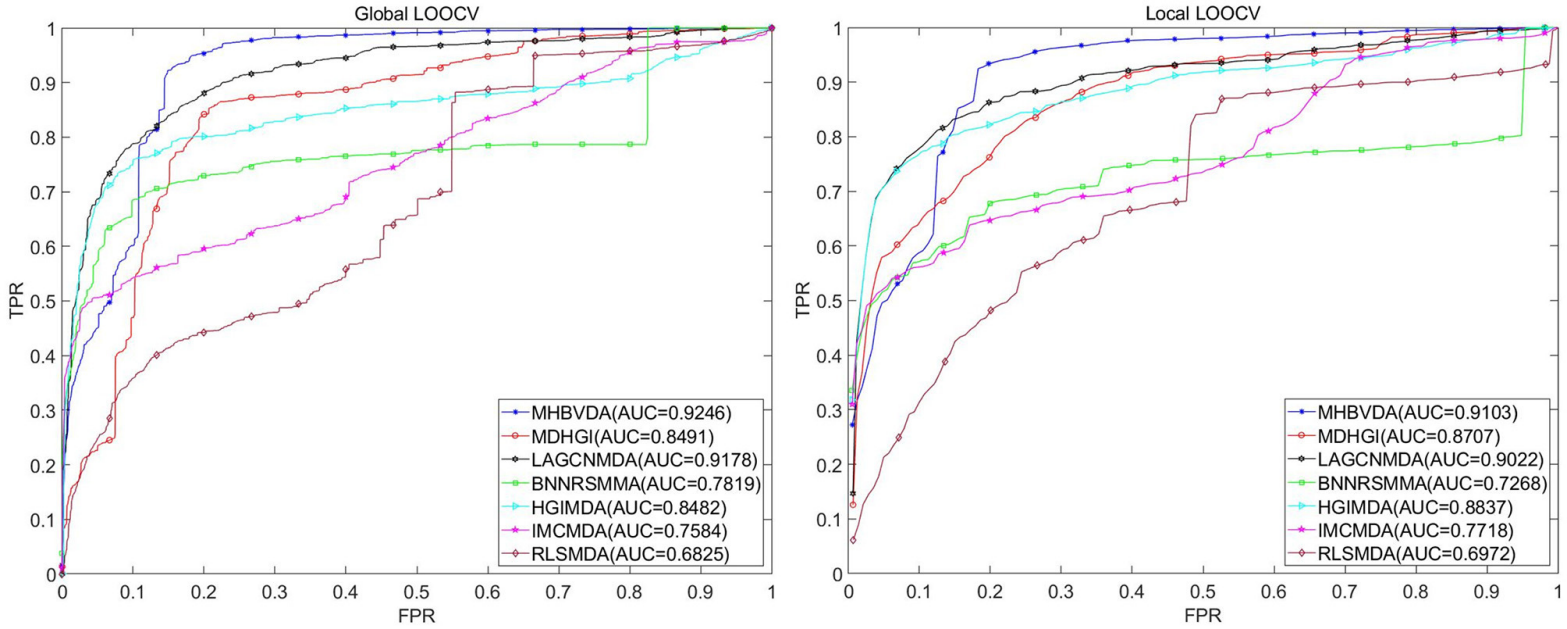
Performance comparison between MHBVDA and previous five association prediction models (MDHGIMDA, LAGCNMDA, BNNRSMMA, HGIMDA, IMCMDA, and RLSMDA) in AUC values of global LOOCV **(left)** and local LOOCV **(right)** based on the aBiofilm dataset.

The dataset of aBiofilm includes 2,884 drug–microbe associations that consist of 1,720 drugs and 140 microbes obtained from the aBiofilm database (https://bioinfo.imtech.res.in/manojk/abiofilm/) (Rajput et al., 2018). For the dataset of aBiofilm, in the global LOOCV, MHBVDA derived better performance with an AUC of 0.9246 than AUCs of HGIMDA (0.8482), IMCMDA (0.7584), RLSMDA (0.6825), BNNRSMMA (0.7819), MDHGI (0.8491), and LAGCNMDA (0.9178) (see Figure 4). In the local LOOCV, MHBVDA obtained an AUC of 0.9103, which is better than AUCs of HGIMDA (0.8837), IMCMDA (0.7718), RLSMDA (0.6972), BNNRSMMA (0.7268), MDHGI (0.8707), and LAGCNMDA (0.9022) (see Figure 4). In the 5-fold cross-validation, AUCs and standard deviations of HGIMDA (0.8412 ± 0.0014), IMCMDA (0.7509 ± 0.0073), RLSMDA (0.6338 ± 0.0140), BNNRSMMA (0.7340 ± 0.0073), and MDHGI (0.8176 ± 0.0026) are less than AUCs and standard deviations of MHBVDA (0.8871 ± 0.0021) (see Table 3). Similarly, although the AUCs and standard deviations of LAGCNMDA (0.9141 ± 6.8556e-04) are higher than those of MHBVDA, the global LOOCV and local LOOCV results of MHBVDA are higher than those of LAGCNMDA.

The results showed that AUCs of LOOCV for MHBVDA are higher than those of other compared algorithms based on MDAD and aBiofilm datasets. However, AUCs of 5-fold cross-validation for MHBVDA are less than LAGCNMDA based on MDAD and aBiofilm datasets. The outcome occurs because LAGCNMDA is a deep learning-based model for which a huge amount of training data is usually required. The size of the MDAD and aBiofilm datasets is larger than the DrugVirus dataset, which may cause LAGCNMDA to have a higher AUC than MHBVD in 5-fold cross-validation.

#### Statistical significance report on AUC values

We further evaluated the significance of performance differences between MHBVDA and BNNRSMMA by using paired *t-*tests. For the DrugVirus dataset, the results showed that the *p*-values between MHBVDA and BNNR were 0.9591 and 0.9008 based on global and local LOOCV, respectively. That is because DrugVirus is a small sample dataset with only 933 known virus–drug associations, so there is no significant difference between MHBVDA and BNNRSMMA on the DrugVirus dataset. However, for the MDAD dataset, the *p*-values between MHBVDA and BNNR were 2.7357e-14 and 2.0389e-04 based on global and local LOOCV, respectively. For the aBiofilm dataset, the *p*-values between MHBVDA and BNNR were 6.2742e-45 and 4.5957e-10 based on global and local LOOCV, respectively. The results showed that MHBVDA is significantly different from BNNRSMMA based on the datasets of MDAD and aBiofilm.

### Discussing parameters of model

It is worth mentioning that MHBVDA is an integration model based on MDHGI and BNNR by using ensemble learning. The weight of MDHGI and BNNR *w*_2_ would affect the performance of MHBVDA. To obtain better performance while ensuring that the sum of *w*_1_ and *w*_2_ is equal to 1, we tested nine groups of weights of and *w*_2_ with a range from 0.1 to 0.9 (step size 0.1), based on the global LOOCV of the DrugVirus, MDAD, and aBiofilm datasets, respectively. Subsequently, we selected the best performance weights from the tested nine groups for any of the three datasets and applied them to 5-fold cross-validation and local LOOCV. The result showed that the weights of MDHGI and BNNR are set at 0.3 and 0.7 (0.9 and 0.1, 0.9 and 0.1) for DrugVirus (MDAD, aBiofilm).

### Case studies

Two types of case studies were further implemented to validate the prediction ability of the MHBVDA. In the first type of case study, ZIKV, SARS-CoV-2, and HIV-1 from the DrugVirus dataset were chosen as investigated viruses, respectively. We ranked the investigated virus–drug pairs that have an unknown association in descending order according to the scores predicted by MHBVDA. Then, the number of the top 50 investigated virus–drug associations would be confirmed by the literature. In the second type of case study, *Pseudomonas aeruginosa* from the MDAD dataset was chosen as the investigated microbe. We removed all known associated drugs for *Pseudomonas aeruginosa*. Then, we ranked the investigated microbe–drug pairs that have an unknown association in descending order according to the scores predicted by MHBVDA. Finally, the number of the top 50 investigated microbe–drug associations would be confirmed by the literature and MDAD dataset. It is worth mentioning that the prediction results presented in the case study were validated by databases and published literature. For some predicted associations that have not yet been validated through existing literature and databases, it is our hope that biologists will conduct biological experiments to further confirm them in the future.

ZIKV was first isolated from non-human primates in 1947 (Musso and Gubler, 2016). According to the study (Musso and Gubler, 2016), ZIKV belongs to the Flaviviridae family and is usually spread by mosquitoes. In addition, ZIKV infection could cause sporadic febrile illness (Musso and Gubler, 2016). So far, cases of ZIKV infection have been reported in Southeast Asia, South America, North America, and other regions, posing a huge threat to global public health (Wikan and Smith, 2016). In this case, through the implementation of MHBVDA, ZIKV-related drugs would be identified. Then, we ranked ZIKV-related drugs in descending order according to the scores predicted by MHBVDA. At last, the top 50 ZIKV–drug associations would be confirmed by searching the literature on PubMed. The results showed that 19 out of the top 50 drugs for ZIKV were confirmed (see Table 4). For example, the predicted result showed that associations between ZIKV and Labyrinthopeptin A1 (Laby A1) ranked third. Laby A1 is a prototype peptide of carbacyclic lantibiotics and has antiviral activity for HIV (Férir et al., 2013). Oeyen et al. (2021) found that Laby A1 can inhibit infection with ZIKV by employing time-of-drug addition experiments. The association between ZIKV and chlorpromazine was predicted and ranked sixth. Chlorpromazine was synthesized in 1951 and used as a potentiator of general anesthesia in 1952 (Ban, 2007). Persaud et al. (2018) demonstrated that chlorpromazine can inhibit ZIKV in host cells by using a cell viability assay.

**Table 4:** The top 50 predicted drugs against ZIKV.

**Drug name**	**Evidence**	**Drug name**	**Evidence**
Didanosine	Unconfirmed	Enfuvirtide	Unconfirmed
Artesunate	Unconfirmed	Irbesartan	Unconfirmed
Labyrinthopeptin A1	PMID: 31666384	Homoharringtonine	PMID: 33231855
Amiodarone	Unconfirmed	Clomipramine	Unconfirmed
Inosine pranobex	Unconfirmed	Camostat	Unconfirmed
Chlorpromazine	PMID: 32210929	4-HPR (Fenretinide)	PMID: 29051080
Doxycycline	PMID: 30576587	Efavirenz	PMID: 32326119
Itraconazole	Unconfirmed	Diphyllin	PMID: 31501074
Azacitidine	PMID: 29698664	Bortezomib	PMID: 28531943
Anisomycin	PMID: 3208140	Atovaquone	PMID: 33371476
Isolanid (lanatoside C)	Unconfirmed	Apoptozole	PMID: 31967789
Ivermectin	PMID: 32942671	Clevudine	Unconfirmed
Erlotinib	Unconfirmed	Emtricitabine	Unconfirmed
Emodin	PMID: 30641880	Entecavir	Unconfirmed
Amprenavir	Unconfirmed	Formoterol	Unconfirmed
Glycyrrhizin	PMID: 33388394	Amantadine	Unconfirmed
Gefitinib	Unconfirmed	Brequinar	PMID: 27919709
Idoxuridine	Unconfirmed	Adefovir Dipivoxil	Unconfirmed
Camptothecin	Unconfirmed	Filociclovir	Unconfirmed
ABT-263	Unconfirmed	Dasatinib	Unconfirmed
Brincidofovir	Unconfirmed	Fluoxetine	Unconfirmed
Foscarnet	Unconfirmed	Fosamprenavir	Unconfirmed
5-Bromovinyldeoxyuridine	Unconfirmed	Benztropine	Unconfirmed
Eflornithine	PMID: 27535047	Favipiravir	PMID: 29425176
Aprotinin	Unconfirmed	Asunaprevir	PMID: 32488021

The first column records the top 25 drugs and the third column records the top 26–50 drugs.

We chose SARS-CoV-2 as the second case. As we all know, the outbreak of SARS-CoV-2 at the end of 2019 posed a huge threat to global public health (Zhu et al., 2020). After the spike protein of SARS-CoV-2 enters cells, SARS-CoV-2 can lead to respiratory lesions and lung damage (V'kovski et al., 2021). In this study, we used MHBVDA to predict potential drugs for SARS-CoV-2. Afterward, we sorted potential drugs associated with SARS-CoV-2 according to predicted scores and verified the top 50 potential drugs for SARS-CoV-2 by finding the literature on PubMed. As a result, 25 out of the top 50 drugs for SARS-CoV-2 were confirmed (see Table 5). Among them, chloroquine was predicted as the fortieth drug against SARS-CoV-2. Chloroquine is an anti-malaria drug that has been used for many years (Touret and De Lamballerie, 2020). Hu et al. (2020) reported that chloroquine may have the potential to treat COVID-19 by studying the absorption of cellular nanoparticles in nanomedicine. Favipiravir was ranked as the forty-fourth potential anti-SARS-CoV-2 drug. Favipiravir is a broad-spectrum inhibitor of viral RNA-dependent RNA polymerase and has been approved as an anti-influenza drug in Japan (Doi et al., 2020). Shannon et al. (2020) found that Favipiravir could be inserted into the RNA of SARS-CoV-2 and could slow RNA synthesis.

**Table 5:** The top 50 predicted drugs against SARS-CoV-2.

**Drug name**	**Evidence**	**Drug name**	**Evidence**
Didanosine	Unconfirmed	Darunavir	PMID: 32889701
Aciclovir	Unconfirmed	Bortezomib	PMID: 32443911
Labyrinthopeptin A1	Unconfirmed	Bevirimat	Unconfirmed
Glycyrrhizin	PMID: 33041173	Cidofovir	Unconfirmed
Amiodarone	PMID: 32737841	Isolanid (lanatoside C)	Unconfirmed
Doxycycline	PMID: 35602049	Fluoxetine	PMID: 33723270
Berberine	PMID: 32656311	4-HPR (Fenretinide)	PMID: 32471278
Inosine pranobex	PMID: 33339426	Emtricitabine	PMID: 30903610
Azacitidine	PMID: 33103107	Fosamprenavir	PMID: 17547501
Alisporivir	PMID: 32376613	Enfuvirtide	Unconfirmed
Amprenavir	PMID: 32607508	Benztropine	Unconfirmed
Erlotinib	Unconfirmed	Diphyllin	PMID: 33668694
Ivermectin	PMID: 32513289	Bepridil	PMID: 32511370
Eflornithine	Unconfirmed	Brequinar	PMID: 36041646
Anisomycin	Unconfirmed	Chloroquine	PMID: 32251731
EIPA (amiloride)	Unconfirmed	Dasatinib	PMID: 32661494
Idoxuridine	Unconfirmed	Entecavir	Unconfirmed
Hexachlorophene	Unconfirmed	Brecanavir	Unconfirmed
ABT-263	Unconfirmed	Favipiravir	PMID: 32511380
Emodin	PMID: 32194980	Apoptozole	Unconfirmed
Foscarnet	Unconfirmed	Artesunate	PMID: 33153922
Clevudine	Unconfirmed	Efavirenz	Unconfirmed
Irbesartan	Unconfirmed	Adefovir Dipivoxil	PMID: 34440200
Amantadine	PMID: 33804989	5-Bromovinyldeoxyuridine	Unconfirmed

The first column records the top 25 drugs and the third column records the top 26–50 drugs.

HIV-1 was chosen as the third case. HIV-1, a member of the genus Lentivirus in the family Retroviridae, is the pathogen of AIDS (Barré-Sinoussi, 1996). HIV-1 could integrate the proviral genome into chronically infected cells and evolve rapidly during viral replication (Ferguson et al., 2002). Therefore, HIV-1 could lead to sustained infection (Ferguson et al., 2002). Similarly, we employed MHBVDA to predict new drugs for HIV-1. Subsequently, we ranked the top 50 drugs according to predicted scores. The result indicated that 24 out of the top 50 anti-HIV-1 drugs were reported by searching the literature on PubMed (see Table 6). For example, the association between HIV-1 and Berberine was predicted and ranked fourth. Berberine, an isoquinoline alkaloid, has strong pharmacological activity (Och et al., 2020). Shao et al. (2020) found berberine can inhibit HIV-1 entry by blocking HIV-1 cell–cell fusion by employing the Luciferase Assay System, colorimetric XTT assay, and a control experiment. Moreover, the association between HIV-1 and Chloroquine was ranked forty-fourth. The study results of Naarding et al. (2007) suggested that chloroquine may reduce HIV-1 transmission or replication in the body through a variety of mechanisms, including modulation of the gp120 structure. Similarly, the experiment of Savarino et al. (2001) showed that chloroquine could inhibit the replication of HIV-1 by affecting the post-transcriptional production of gp120.

**Table 6:** The top 50 predicted anti-HIV-1 drugs.

**Drug name**	**Evidence**	**Drug name**	**Evidence**
Amiodarone	Unconfirmed	Efavirenz	PMID: 31339676
BCX4430 (Galidesivir)	Unconfirmed	Azacitidine	PMID: 26833151
Chlorpromazine	Unconfirmed	Calanolide A	PMID: 18717330
Berberine	PMID: 32897426	Gefitinib	Unconfirmed
Doxycycline	Unconfirmed	Dasatinib	PMID: 31476293
Didanosine	PMID: 17600392	Eflornithine	Unconfirmed
4-HPR (Fenretinide)	PMID: 16375981	Glycyrrhizin	PMID: 16053446
EIPA (ethylisopropyl amiloride)	Unconfirmed	Brecanavir	PMID: 17620375
Inosine pranobex	Unconfirmed	Itraconazole	Unconfirmed
Labyrinthopeptin A1	PMID: 23724015	Diphyllin	Unconfirmed
Anisomycin	Unconfirmed	Camptothecin	PMID: 27825797
Amprenavir	PMID: 28956865	Indinavir	PMID: 32057044
Alisporivir	PMID: 23524389	Bepridil	Unconfirmed
ABT-263	Unconfirmed	Fluoxetine	Unconfirmed
Enfuvirtide	PMID: 30811933	5-Bromovinyldeoxyuridine	Unconfirmed
Kasugamycin	Unconfirmed	Flavopiridol	PMID: 10906320
Bevirimat	PMID: 19024627	Amantadine	Unconfirmed
Isolanid (lanatoside C)	Unconfirmed	Entecavir	PMID: 17582071
Ivermectin	PMID: 22417684	Chloroquine	PMID: 11166661
Foscarnet	PMID: 9593458	Homoharringtonine	Unconfirmed
Emodin	PMID: 21922907	Benztropine	Unconfirmed
Irbesartan	Unconfirmed	Famciclovir	Unconfirmed
Bortezomib	PMID: 30645648	Brincidofovir	Unconfirmed
Hexachlorophene	Unconfirmed	Clomipramine	Unconfirmed
Apoptozole	Unconfirmed	Ganciclovir	PMID: 28657962

The first column records the top 25 drugs and the third column records the top 26–50 drugs.

*Pseudomonas aeruginosa* is a gram-negative rod-shaped bacterium that causes many diseases in humans (Skariyachan et al., 2018). Particularly, *Pseudomonas aeruginosa* colonizes cystic fibrosis patients' lungs and is responsible for decreased respiratory function (Camus et al., 2021). In the model, we used MHBVDA to predict potential drugs for *Pseudomonas aeruginosa* by removing all known associated drugs for *Pseudomonas aeruginosa* from the MDAD dataset. Afterwards, we sorted potential drugs associated with *Pseudomonas aeruginosa* according to predicted score and verified the top 50 potential drugs for *Pseudomonas aeruginosa* by finding the literature on PubMed and the MDAD dataset. As a result, 22 out of the top 50 drugs for *Pseudomonas aeruginosa* were confirmed (see Table 7). For example, the association between *Pseudomonas aeruginosa* and penicillic acid was predicted and ranked first. Penicillic acid is a polyketide mycotoxin produced by several species of Aspergillus and Penicillium (Sorenson and Simpson, 1986). Liaqat et al. (2010) found that in *Pseudomonas aeruginosa*, the biofilm formation ability of *Pseudomonas aeruginosa* was enhanced with an increase in Penicillic acid concentration. Furthermore, the association between *Pseudomonas aeruginosa* and Betulin was ranked fourth. Rajkumari et al. (2018) found that at sublethal concentrations, Betulin attenuated the production of *Pseudomonas aeruginosa* virulence factors and biofilm formation by affecting the quorum sensing regulatory system of *Pseudomonas aeruginosa*.

**Table 7:** The top 50 predicted associated drugs for *Pseudomonas aeruginosa*.

**Drug name**	**Evidence**	**Drug name**	**Evidence**
Penicillic acid	PMID: 20111864	Virginiamycin M1	Unconfirmed
Patulin	PMID: 34990945	Pa-MAP	Unconfirmed
Avibactam	MDAD	Dalfopristin	Unconfirmed
Betulin	PMID: 29526565	Pleurocidin	MDAD
Phenanthrene	PMID: 32871516	Aloe Vera Gel	PMID: 26266047
Undecanoic acid	Unconfirmed	Vidarabine	Unconfirmed
Myristic acid	MDAD	Metronidazole	Unconfirmed
Epigallocatechin Gallate	PMID: 27784787	C6-HSL	PMID: 34411944
4-chloroquinoline	Unconfirmed	Genistein	Unconfirmed
7-chloroquinolin-4-ol	Unconfirmed	Sorivudine	Unconfirmed
Butane-1,4-diamine	Unconfirmed	LL-37	PMID: 21772832
Ethane-1,2-diamine	Unconfirmed	Hinokitiol	Unconfirmed
N1-(7-chloroquinolin-4-yl)dodecane-1,12-diamine	MDAD	Virstatin	Unconfirmed
N1-(7-chloroquinolin-4-yl)ethane-1,2-diamine	Unconfirmed	N-Acetylcysteine	PMID: 33477393
N1-(7-chloroquinolin-4-yl)octane-1,8-diamine	MDAD	Sodium Chloride	Unconfirmed
N1-[7-(trifluoromethyl)quinolin-4-yl]dodecane-1,12-diamine	MDAD	Terpinene-4-ol	Unconfirmed
N1-[7-(trifluoromethyl)quinolin-4-yl]ethane-1,2-diamine	Unconfirmed	Curcumin	PMID: 32765020
N1-[7-(trifluoromethyl)quinolin-4-yl]octane-1,8-diamine	Unconfirmed	C4-HSL	PMID: 30779754
N-Nonanoyl-cyclopentylamide	Unconfirmed	Clarithromycin	PMID: 30267753
octane-1,8-diamine	Unconfirmed	Scrambled LL-37	Unconfirmed
Phenol,2,4-bis(1,1-dimethylethyl)	Unconfirmed	Palivizumab	Unconfirmed
Quinolin-8-ol	Unconfirmed	DispersinB-KSL-W wound gel	PMID: 24445333
Vancomycin	PMID: 26980934	Albendazole	PMID: 20726341
Sorbitol	Unconfirmed	2(5H)-furanone	Unconfirmed
Raspberry KAS 434	MDAD	Clofazimine	Unconfirmed

The first column records the top 25 drugs and the third column records the top 26–50 drugs.

In addition, to further evaluate the reliability of the prediction performance for MHBVDA, according to previous experience (Tang et al., 2021), we have identified the most frequent potential drugs for BK virus by implementing MHBVDA and compared algorithms based on the DrugVirus dataset. As shown in Table 8, Cidofovir, Brincidofovir, and Mycophenolic acid were predicted by five compared algorithms. Artesunate, Rapamycin (Sirolimus), Erlotinib, Topotecan, and Chloroquine were predicted by four compared algorithms. Particularly, the top 20 drugs predicted by MHBVDA for the BK virus have been predicted at least once by the six compared algorithms. The results showed that top-ranked predictive drugs are more important than low-ranked predictive drugs, and compared algorithms are more likely to predict valuable drugs.

**Table 8:** The most frequent potential drugs for the BK virus predicted by using the six methods based on the DrugVirus dataset.

**MHBVDA**	**Drugs**	**Statistics**
1	Cidofovir	5
2	Brincidofovir	5
3	Artesunate	4
4	Leflunomide	3
5	Sunitinib	3
6	Rapamycin (Sirolimus)	4
7	Gefitinib	3
8	Mycophenolic acid	5
9	Erlotinib	4
10	Dasatinib	3
11	Imatinib	3
12	Hexachlorophene	3
13	Bithionol	3
14	Topotecan	4
15	Nitazoxanide	2
16	Letermovir	2
17	Ganciclovir	2
18	Saracatinib	1
19	Chloroquine	4
20	Mitoxantrone	1

The first column is the top 20 drug serial numbers predicted by MHBVDA. The second column is the predicted drug name corresponding to the serial number. The third column is the times of the top 20 drugs that MHBVDA predicted were among the top 20 drugs that the six comparison algorithms predicted.

## Discussion

The unexpected outbreak and unrealistic progression of COVID-19 have generated an utmost need to realize promising therapeutic strategies to fight the pandemic. Drug repurposing, an efficient drug discovery technique from approved drugs, is an emerging tactic to face the immediate global challenge (Prasad and Kumar, 2021). It provides a timely and cost-effective method for finding potential therapeutic agents for diseases. Identifying drug–virus associations can not only provide great insight into the understanding of interaction mechanisms between drugs and viruses but also assist in narrowing the screening scope of compound candidates for drug discovery (Long et al., 2021). Considering that traditional experiment methods are time-consuming, laborious, and expensive, computational methods enable the rapid identification of potentially repurposable drug candidates against diseases (Deepthi et al., 2021). In this study, by integrating the dataset of known virus–drug associations, virus sequence similarity, drug chemical structure similarity, drug side effect similarity, and Gaussian interaction profile kernel similarity for drugs and viruses, we developed an ensemble learning model of MHBVDA to predict virus–drug associations based on MDHGI and BNNR. Moreover, we employed LOOCV and 5-fold cross-validation to compare the performance of MHBVDA with the performance of HGIMDA, MCMDA, RLSMDA, BNNRSMMA, MDHGI, and LAGCNMDA based on datasets of DrugVirus, MDAD, and aBiofilm, respectively. The results indicated that MHBVDA obtained better performance than the compared models based on cross-validation. Also, the results of two types of case studies of ZIKV, SARS-CoV-2, HIV-1, and *Pseudomonas aeruginosa* once again proved that MHBVDA has excellent prediction performance.

MHBVDA's outstanding prediction performance is mainly due to the following factors: First, SLM was used to decompose the original virus–drug association matrix into two portions. The first portion is a clean part that is a linear combination of the low-rank matrix and the original virus–drug association matrix. The second portion is noise data, which is a spare matrix. Therefore, we can obtain clean virus–drug association data, which contributes to improving the model's prediction accuracy. Second, the regularization term was incorporated in BNNR, which could reduce the negative effect of the noise data used in the model and effectively solve the overfitting problem. Third, the success of MHBVDA also comes from the integration of several reliable biological data (known virus–drug associations, virus sequence similarity, drug chemical structure similarity, drug side effects similarity, and Gaussian interaction profile kernel similarity for drug and virus). Moreover, many computational models cannot be applied to drugs with no confirmed virus associations or viruses with no confirmed drug associations in the dataset. MHBVDA can be applied to drugs (viruses) for which there are no confirmed virus (drug) associations. Hence, we can implement the model to identify potential drugs for emerging viruses such as SARS-CoV-2.

However, disadvantages also exist with the model. First, the number of known virus–drug associations used in this study is finite, and more experimentally confirmed virus–drug associations will need to be collected in the future. Second, the use of SLM for generating a new virus–drug association matrix may provide unneeded and futile association information. Third, the parameters used in MDHGI and BNNR may not be optimal or even deviations may occur.

Next, we can do some work on the following two aspects. First, some other biological entities such as genes, proteins, disease, and miRNA could be applied to establish a more comprehensive knowledge graph related to drugs and viruses. The embedding of viruses and drugs can be learned by integrating knowledge graphs, aiming to improve the prediction accuracy of the VDA prediction model. Second, since the prediction of association between biological entities is one of the basic tasks in computational biology, MHBVDA can be applied to other related prediction problems, such as drug–drug interaction prediction, microbe–disease association prediction, drug–miRNA association prediction, and miRNA–disease association prediction.

## Data availability statement

The original contributions presented in the study are included in the article/Supplementary material, further inquiries can be directed to the corresponding author.

## Author contributions

JQ led the project, supervised the writing, and did the revision of manuscript. ZS and XC did the experiments, searched the literature, and wrote the manuscript. ZJ and JZ did the subsequent revisions. All authors contributed to the article and approved the submitted version.
